# Split T Cell Tolerance against a Self/Tumor Antigen: Spontaneous CD4^+^ but Not CD8^+^ T Cell Responses against p53 in Cancer Patients and Healthy Donors

**DOI:** 10.1371/journal.pone.0023651

**Published:** 2011-08-12

**Authors:** Takemasa Tsuji, Junko Matsuzaki, Erika Ritter, Anthony Miliotto, Gerd Ritter, Kunle Odunsi, Lloyd J. Old, Sacha Gnjatic

**Affiliations:** 1 Ludwig Institute for Cancer Research Ltd., New York Branch at Memorial Sloan-Kettering Cancer Center, New York, New York, United States of America; 2 Department of Gynecologic Oncology, Roswell Park Cancer Institute, Buffalo, New York, United States of America; Federal University of São Paulo, Brazil

## Abstract

Analyses of NY-ESO-1-specific spontaneous immune responses in cancer patients revealed that antibody and both CD4^+^ and CD8^+^ T cell responses were induced together in cancer patients. To explore whether such integrated immune responses are also spontaneously induced for other tumor antigens, we have evaluated antibody and T cell responses against self/tumor antigen p53 in ovarian cancer patients and healthy individuals. We found that 21% (64/298) of ovarian cancer patients but no healthy donors showed specific IgG responses against wild-type p53 protein. While none of 12 patients with high titer p53 antibody showed spontaneous p53-specific CD8^+^ T cell responses following a single *in vitro* sensitization, significant p53-specific IFN-γ producing CD4^+^ T cells were detected in 6 patients. Surprisingly, similar levels of p53-specific CD4^+^ T cells but not CD8^+^ T cells were also detected in 5/10 seronegative cancer patients and 9/12 healthy donors. Importantly, p53-specific CD4^+^ T cells in healthy donors originated from a CD45RA^−^ antigen-experienced T cell population and recognized naturally processed wild-type p53 protein. These results raise the possibility that p53-specific CD4^+^ T cells reflect abnormalities in p53 occurring in normal individuals and that they may play a role in processes of immunosurveillance or immunoregulation of p53-related neoplastic events.

## Introduction

Increasing evidence shows that both tumor antigen-specific CD4^+^ and CD8^+^ T cells play a critical role in eradicating cancer [1], [2]. We have extensively investigated spontaneous or vaccine-induced immune responses against cancer-testis antigen NY-ESO-1 as a prototype tumor antigen in human [3], [4], [5], [6], [7], [8]. Naturally occurring NY-ESO-1-specific CD4^+^ and CD8^+^ T cell responses were typically detected only in patients who had serum antibody against NY-ESO-1, indicating that spontaneous immune responses against NY-ESO-1 in cancer patients with NY-ESO-1 expressing tumors were highly integrated [3], [4]. Recently, it was shown that after vaccination with NY-ESO-1 protein and CpG, NY-ESO-1-specific CD4^+^ T cells became detectable first, followed by the appearance of antibody and CD8^+^ T cells, suggesting a role for NY-ESO-1-specific CD4^+^ T cells in facilitating antibody and CD8^+^ T cell responses after immunotherapy [8]. In addition, we recently found that vaccination with MAGE-A3 protein induced integrated MAGE-A3-specific antibody, CD8^+^ and CD4^+^ T cell responses [9]. In non-small cell lung cancer patients who received MAGE-A3 protein formulated in the adjuvant system AS02B, CD8^+^ T cell responses were induced only in patients who developed very high titer antibody and strong CD4^+^ T cell responses. However, such correlation between antibody and T cell responses has not been fully addressed for other human tumor antigens.

Over the past decades, immune responses against p53 have been extensively investigated [10], [11], [12], [13]. The p53 protein was discovered in our laboratory as an immunogenic tumor-specific antigen by serological investigation of tumor-bearing mice [14], and concomitantly in two other laboratories using other methods [15], [16]. It was later discovered that the p53 gene is frequently mutated in various cancers, leading to loss of heterozygoty, dysregulation of p53 feedback networks, and ultimately resulting in slower p53 turnover and thus accumulation of mutant p53 protein in tumor cells. In humans, p53 is accumulated in up to 70% of tumors from patients with certain cancers such as colon or head and neck cancer, and this accumulation is a highly immunogenic event, spontaneously triggering high-titered specific antibody responses [12]. Indeed, p53 has been one of the most frequently detected antigens recognized by naturally occurring antibodies in cancer patients by the screening of cDNA expression libraries derived from human tumors with autologous antibody (SEREX) and by ELISA in our laboratory [17], [18], [19], [20]. Naturally occurring p53 serum antibodies in cancer patients are known to recognize the wild-type N-terminus or C-terminus sequences of p53 but not the central region of the protein where 90% of mutations occur. We and others have also investigated T cell responses against wild-type p53 and many epitopes for both CD8^+^ and CD4^+^ T cell have been determined by reverse immunology approaches and repeated stimulation of T cells with synthesized peptides [10], [21], [22]. Recent results of T cell immunomonitoring of ovarian and colorectal cancer patients vaccinated with p53 overlapping peptides showed strong induction of p53-specific CD4^+^ T cell responses, that were even detectable by *ex vivo* analyses of peripheral blood mononuclear cells (PBMCs) after vaccination without inducing any detectable p53-specific CD8^+^ T cells [23], [24].

In the present study, we have investigated spontaneous antibody and T cell responses against p53 in ovarian cancer patients, whose tumors frequently accumulate p53 protein [25], and healthy donors. To compare the *in vivo* immunogenicity of p53 with that of NY-ESO-1, we monitored p53-specific CD4^+^ and CD8^+^ T cell responses using the immunomonitoring procedures that were developed to monitor spontaneous NY-ESO-1-specific T cell responses. These standardized methods were previously shown to detect NY-ESO-1-specific circulating CD4^+^ and CD8^+^ T cell responses only in NY-ESO-1-seropositive patients with NY-ESO-1-expressing tumor, but not in healthy donors with low frequency of precursors [4], [26]. Using this protocol, we found that IFN-γ-producing CD4^+^ T cells against wild-type p53 sequences were detected frequently in seropositive cancer patients. Surprisingly, spontaneous p53-specific CD4^+^ T cell responses of similar magnitude were also found in most seronegative patients and healthy donors. In contrast, no spontaneous CD8^+^ T cell responses against wild-type p53 nor against the patients' own mutated p53 sequences were detected in any donors tested, which suggests that the spontaneous activation of CD8^+^ T cell responses against p53 is strongly controlled *in vivo*.

## Materials and Methods

### Patients and healthy donors' samples

PBMCs and serum were obtained from ovarian cancer patients at the Roswell Park Cancer Institute, under an approved protocol from the Institutional Review Board. Written informed consent was obtained from all patients. PBMCs and serum from healthy donors were obtained from New York Blood Center.

### Measurement of serum p53-specific antibody

Recombinant wild-type p53 was produced and p53-specific serum antibody level was measured as described previously [20]. A reciprocal titer was estimated from optical density readings of serially diluted samples and controls as described [20].

### Overlapping peptides

Sequences of overlapping peptides from wild-type p53 protein used for presensitization and detection are shown in Table S1. All peptides were synthesized by Biosynthesis (Lewisville, TX) and were dissolved in dimethyl sulfoxide (DMSO) at 10 mM concentration and stored at −80°C. They were designed to be 25 amino acid length except #1 (19-mer) and #30 (22-mer) and have 15 amino acid overlaps. Amino acid for codon 72 of wild-type p53 frequently shows Arg to Pro polymorphism [27]. To deal with this polymorphism, two different peptides with Arg or Pro at position 72 were prepared for peptide #6 and were mixed at a 1∶1 ratio.

### Presensitization

*In vitro* presensitization was performed as described before [28]. CD8^+^ and CD4^+^ T cells were separated from PBMCs by using Dynabeads (Invitrogen-Dynal AS) and were independently stimulated with CD8^−^CD4^−^ cells that were pulsed overnight with 1 pool (#1–#30), 2 pools (#1–#15 and #16–#30), or 3 pools (#1–#10, #11–#20, and #21–#30) of overlapping peptides according to the number of cells after separation and irradiated. Cell cultures were supplemented with 10 U/ml IL-2 and 20 ng/ml IL-7 twice a week. In parallel, a portion of CD4^+^ T cells was polyclonally expanded with phytohaemagglutinin (PHA, Remel) in the presence of low dose IL-2 and IL-7, to be used as target cells (T-APC) in ELISPOT assay. In some experiments, CD4^+^ T cells were further separated into CD45RA^+^ and CD45RA^−^ subsets by phycoerythrin (PE)-conjugated anti-CD45RA monoclonal antibody (mAb) (BD Biosciences) and anti-PE-magnetic beads (Miltenyi Biotec).

### Detection of p53-specific T cell response

p53-specific IFN-γ production was evaluated by ELISPOT assay at day 9–12 for CD8^+^ T cells and day 18–25 for CD4^+^ T cells. First, T cells were evaluated for their reactivity against 6 pools of 5 peptides (#1–#5, #6–#10, etc.). In some experiments, a pool of 17 overlapping peptides from NY-ESO-1 was used as irrelevant peptides. If IFN-γ production was detected against any of peptide pools, independent peptides in the pool were tested to determine a single peptide recognized. All results from ELISPOT assays were presented as the average number of IFN-γ spot forming cells from duplicate wells without subtracting the number of background spots against unpulsed target cells. Responses were considered positive if the number of spots against peptide(s)-pulsed target cells was 3 times more than that against unpulsed target cells and more than 25 spots/50,000 effector T cells. For some patients, p53-specific T cells were detected by intracellular cytokine staining and CD107a expression assays. Intracellular cytokine staining for IFN-γ, IL-4, and TNF-α was performed as described previously [28]. For CD107a expression assays, presensitized CD8^+^ T cells were restimulated in the presence of 40 µl/ml PE-conjugated anti-CD107a mAb (BD Biosciences) and 0.66 µl/ml GolgiStop (BD Biosciences). In some experiments, p53 peptide-specific CD4^+^ T cells were isolated by flow-cytometric sorting of CD154 expressing cells after restimulation with p53 peptides and they were polyclonally expanded with PHA as described previously [28]. Recognition of naturally-processed p53 protein and cytokine production by p53-specific CD4^+^ T cell lines were investigated by using autologous monocyte-derived dendritic cells (mo-DCs) pre-pulsed overnight with p53 overlapping peptides (3.3 µM), recombinant p53 protein (20 µg/ml) or control NY-ESO-1 protein (20 µg/ml). Cytokine levels in the supernatants were measured by sandwich ELISA using the following mAb pairs: unlabelled and biotinylated-anti-IFN-γ (BD Biosciences), unlabelled and biotinylated-anti-GM-CSF mAbs (BD Biosciences), unlabelled and biotinylated-anti-IL-4 mAbs (BD Biosciences), unlabelled and biotinylated-anti-IL-13 mAbs (eBioscience), unlabelled and biotinylated-anti-IL-10 mAbs (eBioscience), unlabelled and biotinylated-anti-TGF-β mAbs (eBioscience), unlabelled and biotinylated-anti-IL-17 mAbs (eBioscience), and unlabelled and biotinylated-anti-IL-9 mAbs (eBioscience). Standard cytokine proteins were obtained from eBioscience.

## Results

### Anti-p53 antibody response

**Figure 1:**
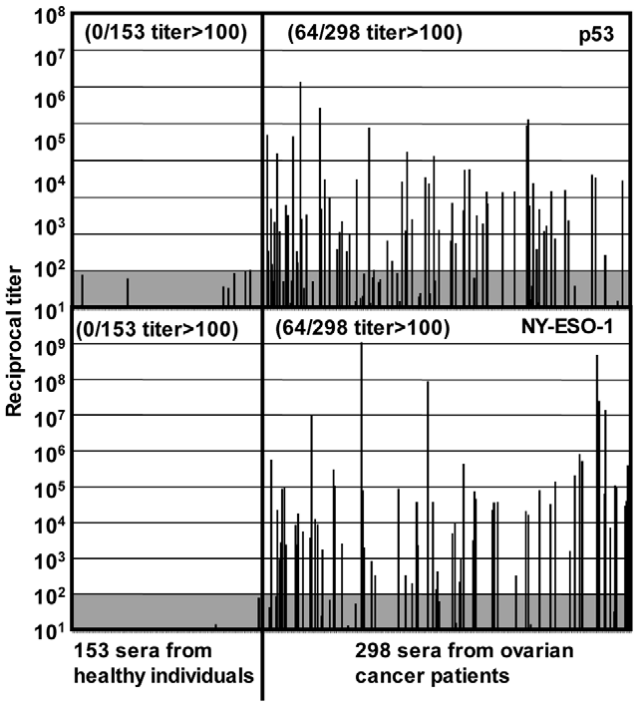
Spontaneous antibody responses in ovarian cancer patients. Sera from 298 ovarian cancer patients and 153 healthy individuals were evaluated for the antibody against wild-type p53 and NY-ESO-1 proteins by ELISA. Reciprocal titers are calculated as described in the reference [20].

To evaluate spontaneous immunity against p53, we measured serum antibody titers against p53 and NY-ESO-1 proteins in 298 advanced epithelial ovarian cancer patients and 153 healthy individuals by ELISA. As shown in Figure 1, 21% of patients but none of the healthy individuals showed significant anti-p53 serum antibody, which was very similar to the frequency of anti-NY-ESO-1 antibody responses observed (21%). These frequencies of spontaneous antibody responses against p53 and NY-ESO-1 are in the range of those reported for ovarian cancer patients in various studies [29]. In contrast, a smaller number of patients (7%) showed significant serum antibody against cancer/testis antigen MAGE-A3 (data not shown). The frequency of patients who showed both NY-ESO-1 and p53 serum antibodies was 4.7%, which is close to the calculated frequency from frequencies against each antigen assuming that the induction of these responses is independent (0.21 (21% against p53)×0.21 (21% against NY-ESO-1) = 0.044 (4.4% against both antigens)).

### Undetectable spontaneous CD8^+^ T cell response in cancer patients and healthy donors

**Figure 2:**
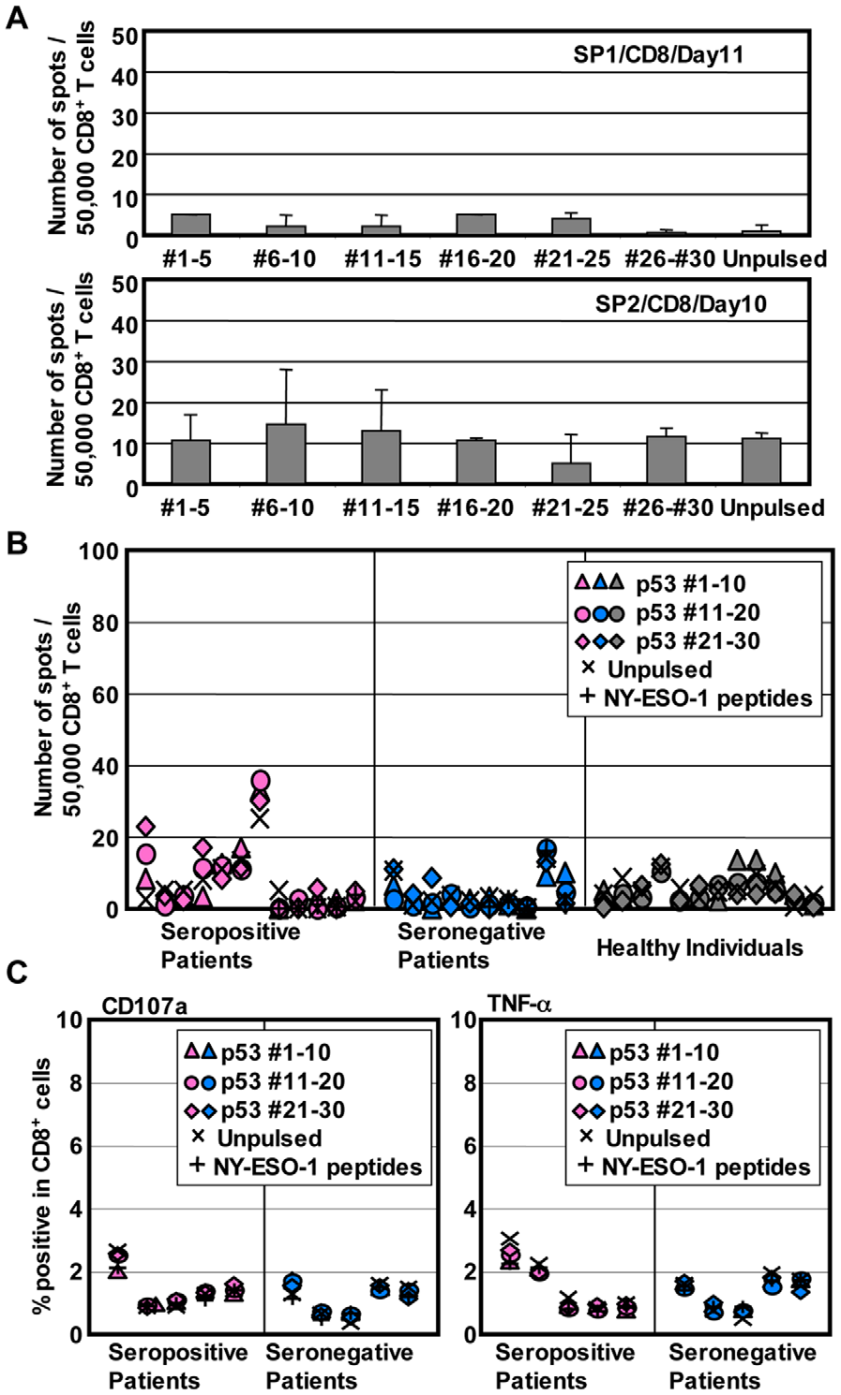
Undetectable p53-specific CD8^+^ T cells in ovarian cancer patients and healthy donors. (A) CD8^+^ T cells from antibody positive ovarian cancer patients were presensitized with a pool of 30 p53 overlapping peptides. After 9–12 days, p53-specific IFN-γ producing T cells were evaluated by ELISPOT assays. Error bars represent standard deviation (SD) of duplicate wells. Autologous T-APCs were used as target cells in ELISPOT assays. (B) CD8^+^ T cell responses in 7 seropositive and 5 seronegative ovarian cancer patients and 12 healthy donors are shown. (C) Presensitized CD8^+^ T cells were analyzed by intracellular TNF-α staining and CD107a expression assays after restimulation with p53-peptides-pulsed, irrelevant (NY-ESO-1) peptides-pulsed or unpulsed target cells.

For the immunomonitoring of spontaneous T cell responses, it is important to select a method that can distinguish *in vivo*-primed T cells from *in vitro*-induced T cells. To monitor spontaneously-induced T cell responses against p53, we employed a single *in vitro* sensitization protocol that has been developed and validated to detect spontaneous NY-ESO-1-specific T cell responses only in NY-ESO-1 seropositive cancer patients but not in healthy individuals. Using this protocol, NY-ESO-1-specific CD8^+^ T cells were frequently detected from NY-ESO-1 seropositive ovarian cancer patients but not from healthy individuals in this study cohort (Figure S1(A)). In contrast, multiple rounds of stimulation by professional antigen processing cells (APCs) such as mo-DCs can induce NY-ESO-1-specific T cells from naive NY-ESO-1-specific precursors in healthy donors [30], [31]. Because of the frequent occurrence of spontaneous serum antibody against p53 in ovarian cancer patients, we first analyzed p53-specific CD8^+^ T cell responses in these patients. From the finding that spontaneous NY-ESO-1-specific T cells are detectable only in seropositive patients, 12 ovarian cancer patients with spontaneous antibody responses against p53 were selected for the evaluation of T cell responses. CD8^+^ and CD4^+^ T cells were isolated from PBMCs as described in materials and methods, and they were separately stimulated with p53 peptide-pulsed CD4^−^CD8^−^ cells. After culturing for about 10 days, the number of IFN-γ-producing p53-specific CD8^+^ T cells was evaluated by ELISPOT assay. As shown in Figure 2A, two representative seropositive patients showed no detectable anti-p53 CD8^+^ T cell response by our standard immunomonitoring protocol. Similarly, no significant IFN-γ production from CD8^+^ T cells was detected from 10 additional seropositive patients (Figure 2B). Ten seronegative ovarian cancer patients and 12 healthy donors were also tested for their spontaneous CD8^+^ T cell response against p53 and as expected, there was no indication of p53-specific CD8^+^ T cells (Figure 2B). It is possible that p53-specific CD8^+^ T cells lack IFN-γ-producing ability and that they are detectable by the expression of other molecules. To test if p53-specific CD8^+^ T cells were detectable by other activation-induced molecules, 5 seropositive and 5 seronegative patients were analyzed for TNF-α production and CD107a expression after restimulation with peptides. As shown in Figure 2C, no p53-specific CD8^+^ T cell response was detected based on TNF-α and CD107a expression.

These results indicate that spontaneous IFN-γ-producing CD8^+^ T cell responses against wild-type p53 were not detectable even in seropositive cancer patients by our presensitization protocol in contrast to the frequent detection of NY-ESO-1-specific CD8^+^ T cell responses in NY-ESO-1-seropositive patients (Figure S1(A)). Accumulation of mutated p53 protein in tumor cells is known to correlate with antibody response [32]. Because mutated peptides are considered to be immunogenic due to absence of tolerance and immunogenicity of mutant p53 to induce CD8^+^ T cell responses was demonstrated in mice [33], [34], we next sought CD8^+^ T cell responses against mutated p53. To do this, exons 5–7 of the p53 gene from tumor tissues and normal lymphocytes of ovarian cancer patients were sequenced to identify potential p53 mutations in the tumor (data not shown). Among the mutations detected, three frequently occurring mutations, S127F, R273C, and R282W, were selected to look for specific CD8^+^ T cell response against epitopes encompassing these mutations. CD8^+^ T cells from three patients who had tumor with S127F, R273C or R282W mutation were stimulated with peptides containing the patient's p53 mutation or with peptides containing the corresponding wild-type sequence. No mutated peptide-specific CD8^+^ T cell response was detected in these three patients (data not shown).

### CD4^+^ T cell responses in cancer patients and healthy individuals

**Figure 3:**
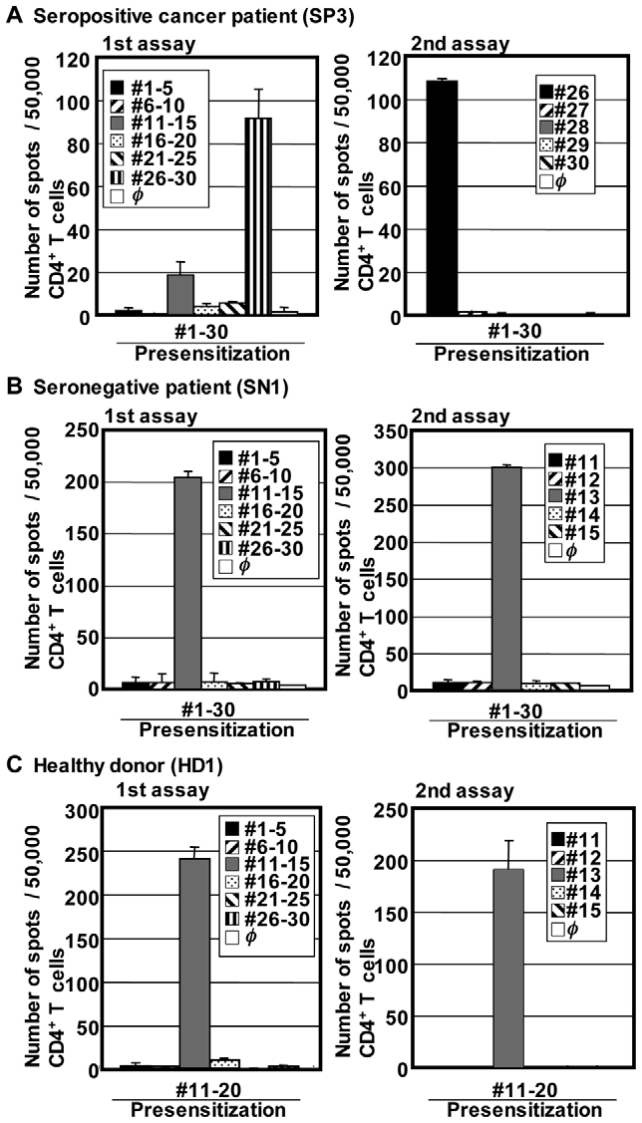
Detection of p53-specific CD4^+^ T cells in ovarian cancer patients and healthy donors. CD4^+^ T cells from antibody positive (A) and negative (B) ovarian cancer patients and healthy donors (C) were presensitized with pool(s) of p53 overlapping peptides. After 18–25 days, p53-specific IFN-γ producing T cells were evaluated against peptide-pulsed or unpulsed (*φ*) target cells by ELISPOT assays. Error bars represent SD of duplicate wells. Autologous T-APCs were used as target cells in ELISPOT assays. T cell responses were first evaluated against subpools of peptides (1st assay) and then against independent peptide in a reactive subpool (2nd assay). For the healthy donor shown in (C), CD4^+^ T cells that were presensitized with #1–#10 or #21–#30 peptide did not show any significant responses (data not shown).

To detect naturally occurring CD4^+^ T cell responses against p53, CD4^+^ T cells from seropositive cancer patients were stimulated with overlapping peptides pulsed on autologous T cell-depleted PBMCs used as APCs. After 20 days of culture in the presence of low-dose IL-2 and IL-7, IFN-γ-producing CD4^+^ T cells were enumerated by ELISPOT assay against subpools of overlapping peptides and then against individual peptides from reactive subpools. In contrast to the undetectable p53-specific CD8^+^ T cells, significant IFN-γ-producing p53-specific CD4^+^ T cell responses to subpools of overlapping peptides were found in 6 out of 12 seropositive patients (Figure 3A and data not shown), with comparable magnitude to spontaneous CD4^+^ T cell responses against NY-ESO-1 in NY-ESO-1 seropositive ovarian cancer patients in this study cohort (Figure S1(B)). We next evaluated spontaneous CD4^+^ T cell responses against p53 in 10 seronegative patients and 12 healthy individuals. Surprisingly, although our presensitization method did not detect NY-ESO-1-specific CD4^+^ T cells in healthy individuals (Figure S1(B)), the same procedure detected significant IFN-γ-producing CD4^+^ T cell responses in 5/10 seronegative patients and 9/12 healthy individuals tested (Figures 3B and C and data not shown). In selected patients, p53-specific CD4^+^ T cells were detectable by TNF-α production following intracellular cytokine staining (Figure S2).

**Figure 4:**
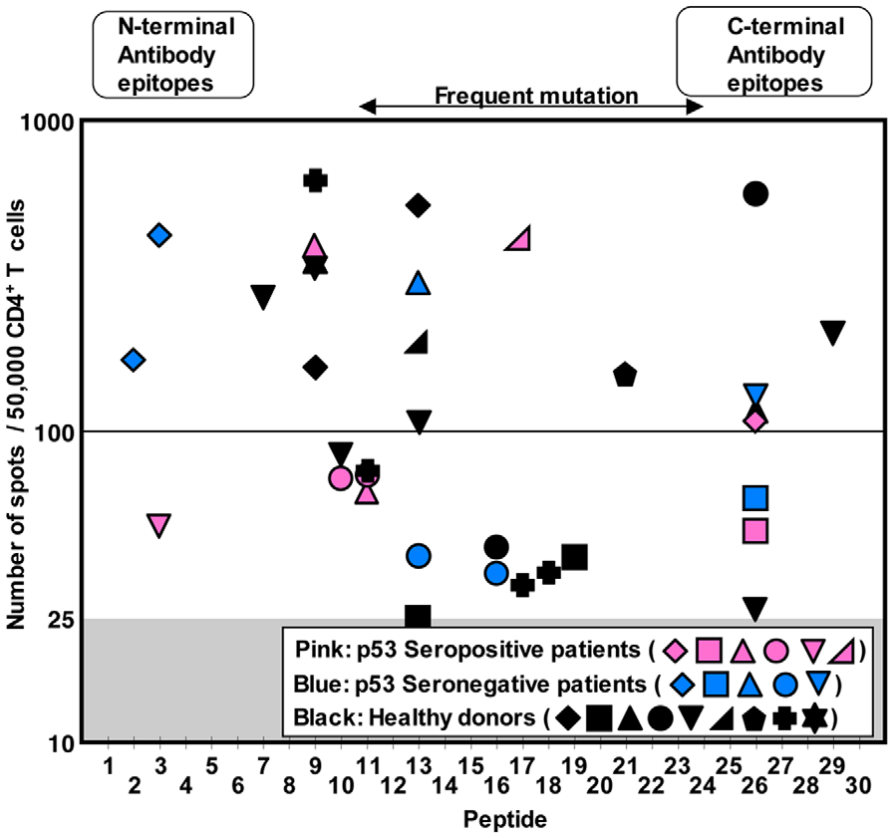
Distribution of CD4^+^ T cell epitopes in ovarian cancer patients and healthy donors. The magnitude of CD4^+^ T cell responses in ovarian cancer patients with or without p53-specific serum antibody and healthy donors against p53 overlapping peptides is plotted. Each symbol represents one individual. All responses shown were significant as compared to the number of background spots. 6/12 seropositive (pink symbols) and 5/10 seronegative (blue symbols) ovarian cancer patients and 9/12 healthy individuals (black symbols) showed positive CD4^+^ T cell responses against p53 peptide-pulsed target cells compared with unpulsed target cells.

Figure 4 summarizes epitopes recognized by CD4^+^ T cells from seropositive and seronegative cancer patients and healthy donors. As shown in Figure 4, epitopes found in both seropositive and seronegative cancer patients and healthy donors were distributed over the entire sequence of the p53 protein. Certain peptides such as #9, #13 and #26 induced more frequent CD4^+^ T cell responses in 4 (20% of responders), 6 (30%) and 7 (35%) donors, respectively. Some seronegative cancer patients and healthy donors showed multiple epitope-specific CD4^+^ T cell responses, as seen in seropositive cancer patients. To consider the frequently observed polymorphism at a codon 72, we used a mixture of two variant peptides for peptide #6 (Table S1), that could induce *de novo* responses in individuals with a p53 homozygous genotype at a codon 72. However, no T cell response against #6 peptides was detected in this study, indicating that our short-term culture protocol did not appear to induce *de novo* CD4^+^ T cell responses. The magnitude of responses and distribution of epitopes were similar in cancer patients and healthy individuals. It is well-known that epitopes recognized by p53-specific antibody are limited to the N-terminus and C-terminus region of the protein [32], [35]. However, there was no correlation between the maps of epitopes for CD4^+^ T cells and for antibodies (Figure 4). In addition, mutation of p53 protein in cancer cells is frequently found in the central region of the gene [32]. However, there was no overrepresentation of CD4^+^ T cell epitopes in this region, suggesting that the responses were not mediated by cross-reactivity of mutated peptide-specific CD4^+^ T cells. To find mutation-specific CD4^+^ T cells in three patients with mutated p53-expressing tumor, CD4^+^ T cells from the patients were presensitized with peptides containing the patients' cognate mutation. Although one patient whose tumor had S127F mutation showed weak CD4^+^ T cell responses against both mutated and corresponding wild-type peptides, no significant mutation-specific CD4^+^ T cell response could be detected (data not shown).

### Origin of repertoire of p53-specific CD4^+^ T cells

**Figure 5:**
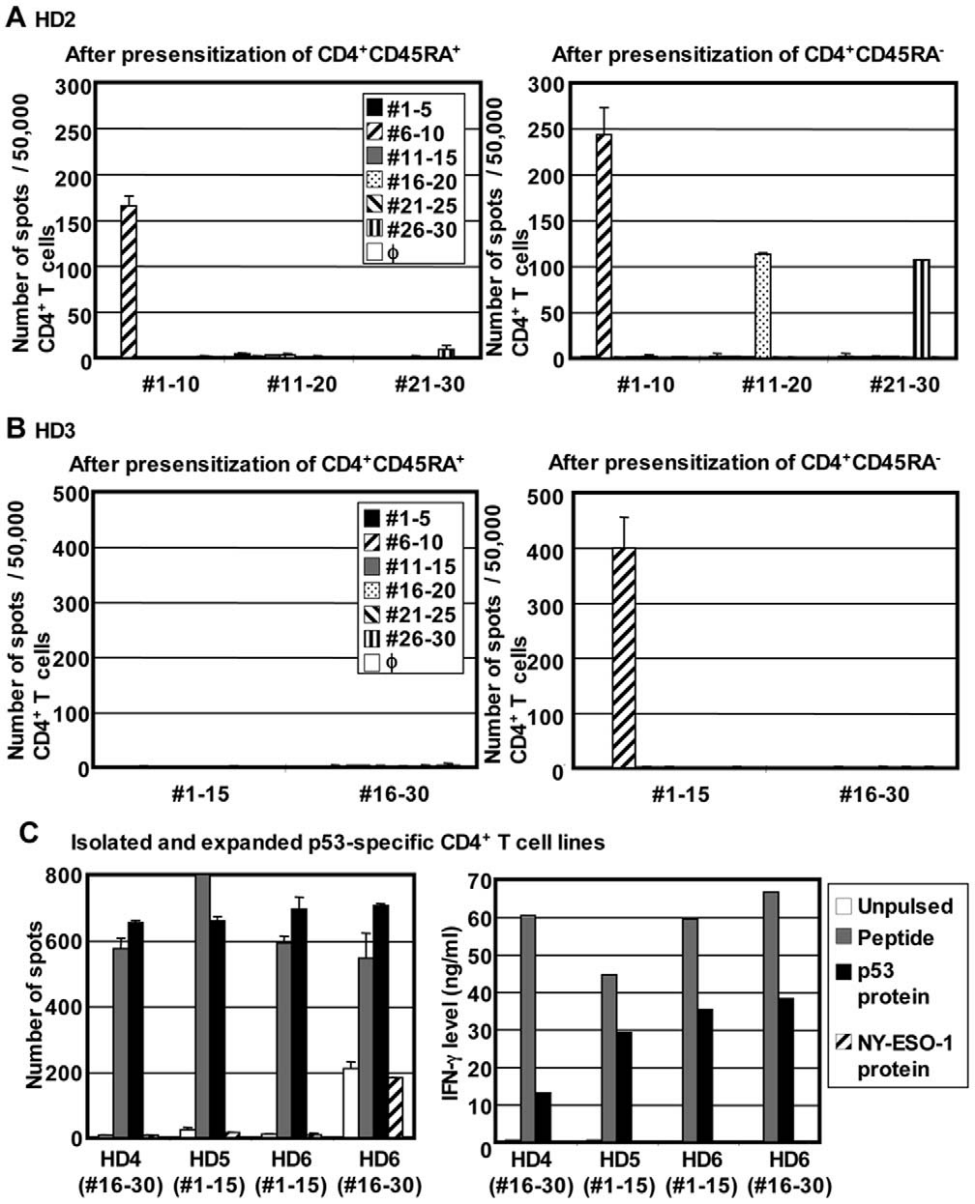
Characterization of p53-specific CD4^+^ T cells in healthy individuals. (A–B) *In vivo* priming of p53-specific CD4^+^ T cells in healthy individuals. CD4^+^CD45RA^+^ naïve and CD4^+^CD45RA^−^ antigen-experienced T cells were isolated by flow cytometry and presensitized with p53 overlapping peptides. After 20 days, p53-specific IFN-γ-producing T cells were evaluated by ELISPOT assays. Autologous T-APCs were used as APCs. (C) Recognition of naturally-processed p53 protein. p53-specific CD4^+^ T cell lines against p53 #1–#15 peptides pool or #16–#30 peptides pool were established from 3 healthy donors as described in materials and methods. They were stimulated by autologous mo-DC pre-pulsed with p53 peptides, p53 protein, or NY-ESO-1 protein. IFN-γ production was evaluated by ELISPOT assays (left) and ELISA (right). Error bars represent SD of duplicate wells.

In contrast to the detection of p53-specific CD4+ T cells in seropositive and seronegative cancer patients as well as in healthy donors, our presensitization protocol only allows detection of NY-ESO-1-specific IFN-γ-producing CD4^+^ T cells in NY-ESO-1 seropositive cancer patients but not in seronegative cancer patients and healthy individuals when PBMC-derived whole CD4^+^ T cells are presensitized with NY-ESO-1 overlapping peptides. However, we recently reported that NY-ESO-1-specific CD4^+^ T cells could be induced from CD45RA^+^ naïve CD4^+^ T cells in healthy donors after *in vitro* presensitization by removing CD25^+^ regulatory T cells [36], [37]. These results indicate that NY-ESO-1-specific CD4^+^ T cell precursors present in healthy individuals are susceptible to suppression by regulatory T cells, but in cancer patients, *in vivo*-primed NY-ESO-1-specific CD4^+^ T cells become resistant to this suppression [36], [37]. Detection of IFN-γ-secreting p53-specific CD4^+^ T cells in healthy individuals after a single *in vitro* sensitization in the presence of regulatory T cells suggests that they are already primed *in vivo*. To determine the activation status of p53-specific CD4^+^ T cells in healthy donors, naïve and antigen-experienced CD4^+^ T cells were separated based on CD45RA expression and were stimulated with p53 overlapping peptides. Induction of p53-specific CD4^+^ T cells was evaluated by ELISPOT assays. As shown in Figures 5A and 5B, in contrast to what had been observed with NY-ESO-1-specific CD4^+^ T cells, p53-specific IFN-γ-producing CD4^+^ T cells were detectable from a CD45RA^−^ antigen-experienced population of healthy donors. In addition, smaller responses were also induced from CD45RA^+^ naïve T cell population (Figure 5A). Because CD45RA is not an absolute marker for naïve T cells, there is still a possibility that p53-specific CD4^+^ T cells in the CD45RA^+^ T cell population were already activated *in vivo* [38]. However, this result indicated that at least a part of p53-specific CD4^+^ T cells have been primed *in vivo* even in healthy individuals.

### Characterization of p53-specific CD4^+^ T cells

To further characterize p53-specific CD4^+^ T cells in healthy donors, p53-specific T cell lines were generated by isolating CD154 expressing CD4^+^ T cells after restimulation with p53 peptides followed by polyclonal expansion with PHA (data not shown). This CD154 expression-based method was employed to detect multiple CD4^+^ T cell subsets for the analysis of cytokine producing pattern [28]. Four CD4^+^ T cell lines were generated against p53 peptide pools from 3 healthy donors. All CD4^+^ T cell lines specifically produced GM-CSF, which is produced from both Th1 and Th2 cells, after stimulation with peptide-pulsed mo-DCs (Table 1). Levels of other cytokines produced by CD4^+^ T cell lines are shown in Table 1. Consistent with the efficient detection of p53-specific CD4^+^ T cells by IFN-γ ELISPOT assays, all p53-specific CD4^+^ T cell lines from healthy donors produced large amounts of IFN-γ. In addition to IFN-γ, significant levels of IL-13 and small amounts of IL-4 were also specifically produced, indicating that the T cell lines were a mixture of Th1 and Th2 cells. Two CD4^+^ T cell lines produced small levels of immunoregulatory cytokine, IL-10. One CD4^+^ T cell line produced small but significant levels of IL-9, which is produced by Th9 and is regulated by IL-4 and TGF-β, potentially indicating the presence of TGF-β signaling during the differentiation of p53-specific CD4^+^ T cells. However, production of TGF-β was not detected in the supernatant (data not shown). In addition, IL-17, which is produced by Th17, was not detected in any CD4^+^ T cell lines (data not shown). Next, the recognition of naturally-processed p53 protein was investigated by using autologous mo-DCs as APCs. As shown in Figure 5C, all CD4^+^ T cell lines efficiently recognized p53 protein-pulsed target cells as detected by IFN-γ secretion. This result demonstrates p53-specific CD4^+^ T cells detectable in healthy donors are able to recognize naturally-processed p53 protein and makes it unlikely that they are activated *in vivo* by other proteins with similar sequences.

**Table 1:** Cytokine levels (ng/ml) in supernatant of isolated and expanded p53-specific CD4^+^ T cell lines of healthy donors.

	IFN-γ	GM-CSF	IL-4	IL-13	IL-10	IL-9
HD4(#16–#30)	**60.1**(0.6)	**8.5**(0.2)	**0.2**(0.0)	**4.9**(0.1)	**0.9**(0.0)	0.0(0.0)
HD5(#1–#15)	**44.4**(0.5)	**8.7**(0.1)	**0.2**(0.0)	**5.0**(0.1)	0.1(0.0)	0.0(0.0)
HD6(#1–#15)	**59.4**(0.3)	**10.9**(0.1)	**0.7**(0.0)	**5.4**(0.1)	0.1(0.0)	**0.1**(0.0)
HD6(#16–#30)	**66.5**(0.1)	**13.4**(0.1)	**0.4**(0.0)	**3.8**(0.0)	**0.8**(0.0)	0.0(0.0)

CD4^+^ T cell lines were obtained from healthy donors by isolating CD154 expressing cells after restimulation with #1–#15 or #16–#30 peptides pool and expanding with PHA. p53-specific CD4^+^ T cell lines (50,000 cells) and peptide-pulsed autologous mo-DCs (50,000 cells) were cocultured for 20 hours and cytokines levels in the supernatant were measured by ELISA. Numbers in parentheses indicate background cytokine production against unpulsed autologous mo-DCs. Cytokine production exceeding the limit of detection and 5 times higher than background production was considered to be significant and is shown in bold.

## Discussion

Accumulation of mutated p53 protein in tumor is observed frequently in many types of tumors and induces humoral immune responses, demonstrating strong immunogenicity of p53 [12]. Thus, p53 has been considered a promising target for vaccination against multiple types of cancer. Yet, we report here the failure to detect *in vivo*-primed p53-specific CD8^+^ T cell responses in cancer patients with spontaneous antibodies to p53 as well as in p53-seronegative patients and in healthy donors using our single *in vitro* sensitization protocol. In contrast, the same protocol frequently detected NY-ESO-1-specific CD8^+^ T cells in NY-ESO-1-seropositive cancer patients in the same study cohort, indicating that natural immunogenicity to induce spontaneous CD8^+^ T cell responses may be different in p53 and NY-ESO-1 even in seropositive patients for these antigens. It was reported that although cyclin B1-specific CD8^+^ T cells became detectable after a single *in vitro* stimulation, p53-specific CD8^+^ T cells against 6 HLA-A*02 binding short peptides could not be detected in 5 cyclin B1-reactive and 5 non-reactive patients using the same method [39]. Our results expand their observations by monitoring CD8^+^ T cell responses to the whole region of the protein using overlapping peptides and by including with immunomonitoring of spontaneous antibody and CD4^+^ T cell responses. Several vaccine trials such as p53-transduced DCs or long overlapping peptides have reported induction of T cell responses with limited clinical benefit [23], [24], [40], [41]. Immunogenicity of p53 in mice was investigated and it was clearly shown that p53-specific CD8^+^ T cells but not CD4^+^ T cells are anergic in wild-type mice [42], [43], [44]. In p53 knock-out mice, immunizing with p53 led to specific CD8^+^ T cells of higher avidity compared to wild-type mice [42]. A study in human leukocyte antigen (HLA)-A*02 transgenic mice demonstrated that unresponsiveness of HLA-A*02-restricted CD8^+^ T cells was limited to the epitopes that were homologous in human and mice, indicating that the tolerance was induced by murine p53 [42], [44]. In contrast, CD4^+^ T cell responses against wild-type p53 were similarly induced and their avidities were almost identical in wild-type and p53^−/−^ mice [43]. The presence of p53-specific CD8^+^ T cell precursors in PBMCs of cancer patients and healthy individuals was reported by several groups and p53-specific CD8^+^ T cells have been found to specifically recognize tumor cells accumulating p53 [22], [45], [46], [47]. However, these tumor-reactive p53-specific CD8^+^ T cells were obtained only after *in vitro* priming and extensive stimulations, and most other p53-specific CD8^+^ T cells from the literature were found to have low avidity [42], [48]. In addition, it was recently shown that p53-specific CD8^+^ T cells detected by *ex vivo* analyses using tetramers express apoptotic markers [49], suggesting that CD8^+^ T cells may fail to expand because of apoptosis following antigen presensitization in our protocol. Wild-type p53 is a ubiquitously-expressed protein but the wild-type p53 protein expression in normal tissue is hardly detectable due to rapid degradation by a proteasome-dependent pathway, which eventually causes antigen presentation on HLA class I molecules [50], [51]. The specificity of CD8^+^ T cells to p53-accumulating tumors was recently investigated, leading to the observation that CD8^+^ T cells transduced with high affinity p53-specific T cell receptor gene could recognize not only p53-accumulating tumors but also tumors expressing wild-type p53, irrespective of the expression level of p53 protein, and normal cells such as peripheral blood stem cells and dendritic cells [52]. Because antigen presentation by professional APCs like DCs in non-inflammatory condition is known to induce tolerance, a constitutive presentation of wild-type p53 by DCs could be considered to induce tolerance of high-avidity p53-specific CD8^+^ T cells [53]. In addition to central tolerance caused by thymic APCs expressing p53 [42], [44], peripheral tolerance induction by p53-expressing APCs may explain our observation that *in vivo*-primed p53-specific CD8^+^ T cells are not detected even in p53 seropositive ovarian cancer patients. It has been reported that p53-specific CD8^+^ T cells exist at low frequency and that they become detectable after multiple stimulations *in vitro* [22], [46], [54]. Although spontaneous activation of p53-specific CD8^+^ T cells is regulated, it is possible that p53-based vaccination or blockade of immune-regulating signals such as CTLA-4 or PD-1 may induce the activation and expansion of p53-specific CD8^+^ T cells.

In contrast to CD8^+^ T cells, p53-specific CD4^+^ T cells were detectable in seropositive cancer patients (Figure 3A). Surprisingly, IFN-γ-producing CD4^+^ T cells against p53 were frequently detected also in seronegative cancer patients and even in healthy donors (Figure 3B and C). Our observation supports the previous finding that p53-specific CD4^+^ T cells were detectable in seronegative colorectal cancer patients [55]. The magnitude and epitope-distribution of CD4^+^ T cell responses were similar in seropositive and seronegative patients and healthy individuals (Figure 4). p53-specific CD4^+^ T cells in healthy donors were of high avidity, recognized naturally-processed p53 protein, and predominantly produced IFN-γ in addition to other Th2-related and immunoregulatory cytokines (Figure 5C and Table 1). In contrast, IFN-γ-producing NY-ESO-1-specific CD4^+^ T cells were detectable after a single presensitization only in seropositive cancer patients but not in seronegative patients and healthy donors. Still, we have recently found that NY-ESO-1-specific CD4^+^ T cells are detectable in healthy donors when CD25^+^ regulatory T cells are removed from the culture during presensitization [37]. Furthermore, we also found that naïve MAGE-A3-specific CD4^+^ T cells in healthy individuals, which were detectable by CD154 expression, were not detectable by IFN-γ ELISPOT assays [28]. The fact that p53-specific CD4^+^ T cells in healthy donors were detectable without the need for depletion of regulatory T cells and they produced IFN-γ after a single presensitization procedure indicated that they were already primed to differentiate into Th1 cells *in vivo*. Indeed, we found that p53-specific CD4^+^ T cells were detectable after expansion from CD45RA^−^ antigen-experienced population. Although CD4^+^CD45RA^+^ cells also contained p53-specific CD4^+^ T cells (Figure 5A), they could be terminally-differentiated effector cells which lack the expression of CCR7 [38]. Further separation of CD45RA^+^ cells into CCR7^+^ and CCR7^−^ subsets is required to analyze the phenotypic distribution of p53-specific CD4^+^ T cells in healthy donors. The fact that p53-specific CD4^+^ T cells were frequently observed in healthy donors and both seropositive and seronegative cancer patients in contrast to CD8^+^ T cells suggested that central and/or peripheral tolerance against CD4^+^ T cells is weak or absent, as indicated by studies in mice [43]. The basis for the induction of *in vivo* priming of wild-type p53-specific CD4^+^ T cells in healthy individuals has not been clarified from the present study. In addition to accumulation of mutated p53 in malignant cells, wild-type p53 can also accumulate in cells in response to cellular stresses such as UV irradiation and nitric oxide and eventually causes cell cycle arrest and cell death by apoptosis [56], [57]. The release of wild-type p53 protein in the presence of inflammatory responses associated with cellular stresses is a possible mechanism to induce p53-specific CD4^+^ T cells in healthy individuals, and it cannot be excluded that frequent p53 mutations not leading to cancer occur in normal individuals. Investigation of the correlation between the presence of p53-specific CD4^+^ T cells and age or risk of cancer development is helpful to understand the mechanism to induce p53-specific CD4^+^ T cell responses. Interestingly, the average age of ovarian cancer patients who showed CD4^+^ T cell responses against p53 was significantly higher than that of patients without CD4^+^ T cell responses (66.9±3.0 (11 responders) vs. 53.8±3.7 (10 non-responders), *p* = 0.013), whereas there was no significant correlation between the average ages of patients and the status of p53-specific serum antibodies (63.9±3.2 (11 seropositives) vs. 57.1±4.4 (10 seronegatives), *p* = 0.221). It is possible that p53-specific B cells were also continuously primed in response to cellular stress-induced p53 release although the response may not be detectable by antibody production. The detection of antibody responses against many tumor antigens is usually associated with clinically evident tumors, although anti-p53 antibody has been found in asbestosis patients before their diagnosis of cancer [58]. The presence of spontaneously activated p53-specific CD4^+^ T cells in healthy individuals is likely to play an important role in rapid induction of antibody responses after malignant transformation.

Similar phenomenon of CD8^+^ T cell selective tolerance induction in the presence of normal CD4^+^ T cell and antibody responses was observed in transgenic mice that express influenza HA protein in the lung and to a lesser extent in the thymus [59]. The tolerance in CD8^+^ T cells was strictly epitope-specific, because these animals could mount normal CD8^+^ T cell responses against an influenza strain carrying a HA epitope with one amino acid substitution from the transgenic HA. If the mechanism for tolerance induction in p53-specific CD8^+^ T cells is similar, it is possible that CD8^+^ T cell responses are raised against mutated p53. We previously demonstrated that one of three mutated p53 sequences that naturally occur in Meth A sarcoma induced CD8^+^ T cell responses in mice [33], [34]. To test this possibility, we attempted to detect mutated peptide-specific CD8^+^ T cells from three patients whose tumor expressed relatively common mutations (S127F, R273C or R282W) by stimulating with the peptide harboring the patient's own mutation. However, no CD8^+^ T cell response against mutated peptides could be detected (data not shown). Because mutated p53 epitopes must have HLA anchoring motifs for the induction of CD8^+^ T cell responses, more patients must be evaluated to demonstrate the presence or absence of mutated p53-specific CD8^+^ T cells and their spontaneous activation in cancer patients. However, it is possible that mutated peptide-specific CD8^+^ T cells are cross-reactive to a corresponding wild-type peptide and also deleted or rendered tolerant by wild-type p53 presented by thymic epithelial cells or peripheral cells like immature DCs.

In summary, this study revealed the strong immunogenicity of p53 in inducing antibody and CD4^+^ T cell responses without inducing CD8^+^ T cell responses. As already revealed from vaccine trials using long overlapping peptides, vaccination with p53 is not expected to induce effective p53-specific CTL responses. In addition, because p53 is an intracellular protein and thus, p53-specific antibody and CD4^+^ T cells are not thought to directly recognize tumor cells by classical antigen presentation pathways, the role of these immune responses in the prevention of cancer is still uncertain. In our preliminary analysis, the presence of anti-p53 CD4^+^ T cells in ovarian cancer patients did not correlate with patients' survival (data not shown), although the number of patients in the analysis is too small to conclude. However, strong immunogenicity of wild-type p53 to induce CD4^+^ T cells suggested that p53 could be utilized as helper epitopes in multivalent cancer vaccine [23]. Although, both NY-ESO-1 and p53 exhibit spontaneous humoral and CD4^+^ T cell immune responses in cancer patients who have antigen-expressing tumors, CD8^+^ T cell responses are undetectable against p53 in contrast to a strong induction of CD8^+^ T cell responses against NY-ESO-1 [3]. From our previous analyses of spontaneous immune responses in cancer patients and healthy individuals, it was found that CD8^+^ T cell responses against MAGE-A3 and NY-CO-58 was restricted compared to NY-ESO-1 (Table S2). In contrast, spontaneous CD4^+^ T cell responses to MAGE-A3 were detected only in seropositive cancer patients but those to NY-CO-58 could be detected even in healthy individuals [9], [28], [60]. Many tumor-related antigens have been discovered and their immunogenicity is being evaluated in relation to their potential target for immunotherapy of cancer [61]. The induction of tumor antigen-specific high avidity CD8^+^ T cells that can efficiently destroy antigen-expressing tumor cells is considered to be critical for successful cancer vaccination. Because both antigen-specific CD4^+^ T cells and antigen-opsonizing antibodies are considered to help in inducing antigen-specific CD8^+^ T cells, each antigen must be evaluated for their antigenic property to induce an integrated immune response or split tolerance.

## Supporting Information

Figure S1 Spontaneous T cell responses against NY-ESO-1 in seropositive ovarian cancer patients and healthy donors. CD8^+^ (A) and CD4^+^ (B) T cells from NY-ESO-1 seropositive ovarian cancer patients and healthy individuals in the same study cohort of p53 immunomonitoring were presensitized with NY-ESO-1 overlapping peptides and NY-ESO-1-specific T cells were evaluated by ELISPOT assays using the same protocol used to monitor T cell responses against p53.
(TIF)

Figure S2 Detection of p53-specific CD4^+^ T cells by intracellular cytokine staining. CD4^+^ T cells from p53 seropositive and seronegative ovarian cancer patients were presensitized with p53 overlapping peptides and p53-specific T cells were evaluated by intracellular cytokine staining of TNF-α and IL-4. (A) Representative staining after co-culture with p53-peptides subpool-pulsed or unpulsed target cells. (B) Summary of cytokine expressing cells in 4 seropositive and 4 seronegative patients.
(TIF)

Table S1 Numbering of overlapping peptides for wild-type p53.
(DOC)

Table S2 Spontaneous immune responses against tumor-related antigens investigated in the New York Branch of Ludwig Institute for Cancer Research.
(DOC)
