# Supplementary figures and images for: Split T Cell Tolerance against a Self/Tumor Antigen: Spontaneous CD4+ but Not CD8+ T Cell Responses against p53 in Cancer Patients and Healthy Donors
Source: PLoS One. 2011 Aug 12;6(8):e23651. doi: 10.1371/journal.pone.0023651 (PMC3155555; doi:10.1371/journal.pone.0023651)

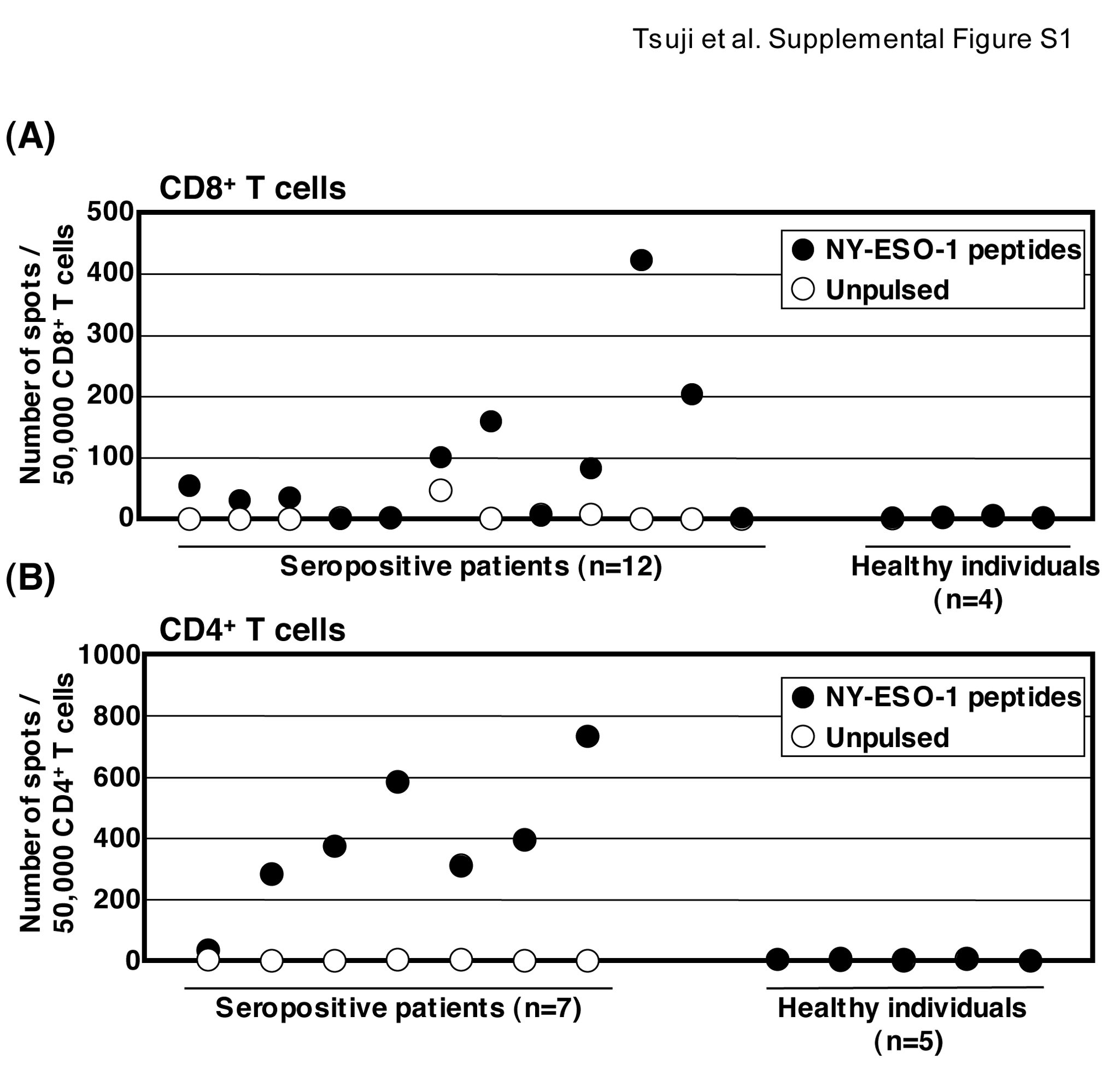

Supplement: Figure S1 — Spontaneous T cell responses against NY-ESO-1 in seropositive ovarian cancer patients and healthy donors. CD8+ (A) and CD4+ (B) T cells from NY-ESO-1 seropositive ovarian cancer patients and healthy individuals in the same study cohort of p53 immunomonitoring were presensitized with NY-ESO-1 overlapping peptides and NY-ESO-1-specific T cells were evaluated by ELISPOT assays using the same protocol used to monitor T cell responses against p53. (TIF) [file pone.0023651.s001.tif]

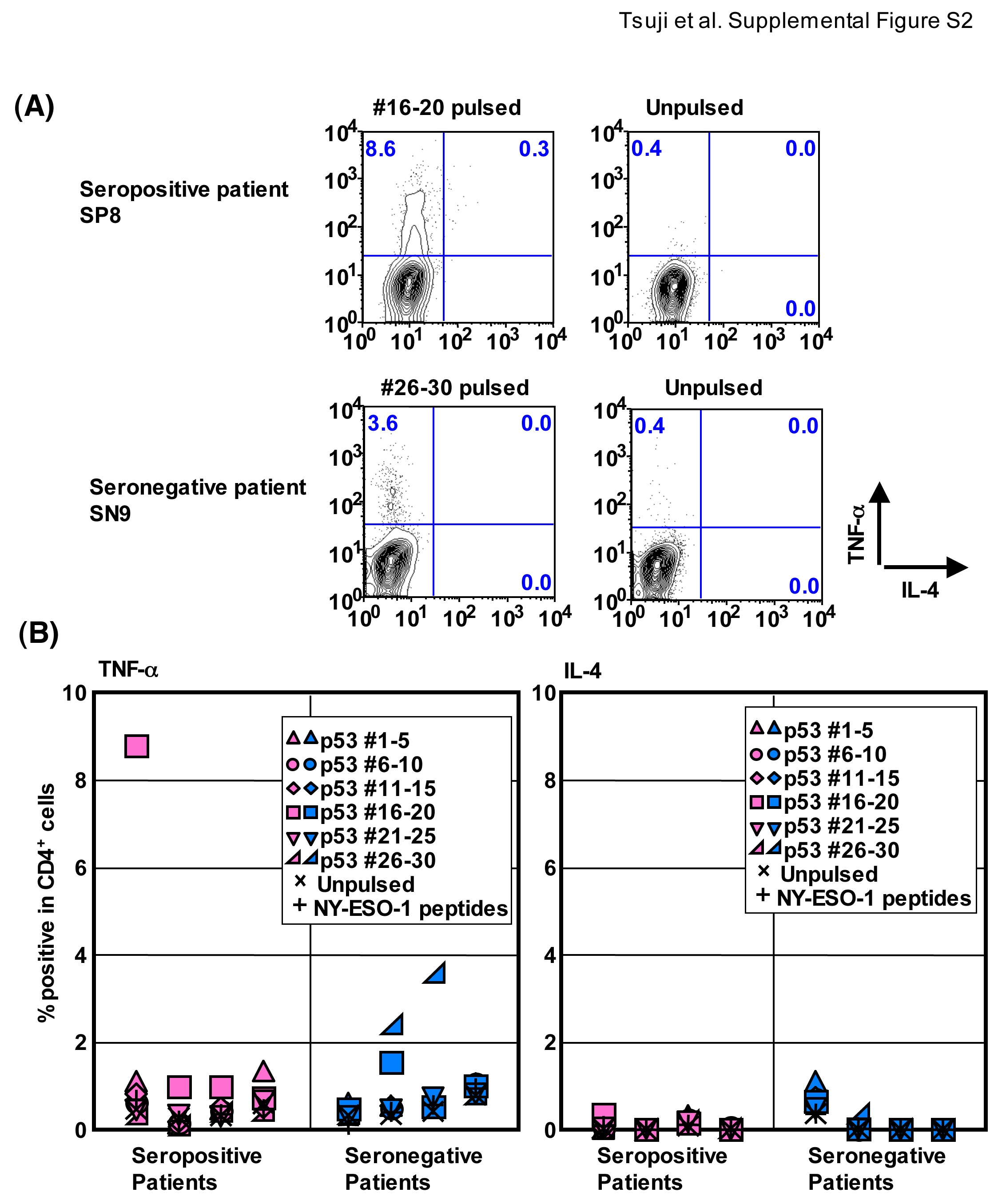

Supplement: Figure S2 — Detection of p53-specific CD4+ T cells by intracellular cytokine staining. CD4+ T cells from p53 seropositive and seronegative ovarian cancer patients were presensitized with p53 overlapping peptides and p53-specific T cells were evaluated by intracellular cytokine staining of TNF-α and IL-4. (A) Representative staining after co-culture with p53-peptides subpool-pulsed or unpulsed target cells. (B) Summary of cytokine expressing cells in 4 seropositive and 4 seronegative patients. (TIF) [file pone.0023651.s002.tif]
