# Supplementary material for: Split T Cell Tolerance against a Self/Tumor Antigen: Spontaneous CD4+ but Not CD8+ T Cell Responses against p53 in Cancer Patients and Healthy Donors
Source: PLoS One. 2011 Aug 12;6(8):e23651. doi: 10.1371/journal.pone.0023651 (PMC3155555; doi:10.1371/journal.pone.0023651)
Supplement: Table S1 — Numbering of overlapping peptides for wild-type p53. (DOC) [file pone.0023651.s003.doc]

**Supplemental Table S1**

Numbering of overlapping peptides for wild-type p53

Number Position Sequence

#1 aa1-19 meepqsdpsvepplsqetf

#2 aa8-32 psvepplsqetfsdlwkllpennvl

#3 aa21-45 dlwkllpennvlsplpsqamddlml

#4 aa34-58 plpsqamddlmlspddieqwftedp

#5 aa47-71 pddieqwftedpgpdeaprmpeaap

#6 aa60-84 pdeaprmpeaap*r*vapapaaptpaa

#6’ aa60-84 pdeaprmpeaap*p*vapapaaptpaa

#7 aa73-97 vapapaaptpaapapapswplsssv

#8 aa86-110 apapswplsssvpsqktyqgsygfr

#9 aa99-123 sqktyqgsygfrlgflhsgtaksvt

#10 aa112-136 gflhsgtaksvtctyspalnkmfcq

#11 aa125-149 tyspalnkmfcqlaktcpvqlwvds

#12 aa138-162 aktcpvqlwvdstpppgtrvramai

#13 aa151-175 pppgtrvramaiykqsqhmtevvrr

#14 aa164-188 kqsqhmtevvrrcphhercsdsdgl

#15 aa177-201 phhercsdsdglappqhlirvegnl

#16 aa190-214 ppqhlirvegnlrveylddrntfrh

#17 aa203-227 veylddrntfrhsvvvpyeppevgs

#18 aa216-240 vvvpyeppevgsdcttihynymcns

#19 aa229-253 cttihynymcnsscmggmnrrpilt

#20 aa242-266 cmggmnrrpiltiitledssgnllg

#21 aa255-279 itledssgnllgrnsfevrvcacpg

#22 aa268-292 nsfevrvcacpgrdrrteeenlrkk

#23 aa281-305 drrteeenlrkkgephhelppgstk

#24 aa294-318 ephhelppgstkralpnntssspqp

#25 aa307-331 alpnntssspqpkkkpldgeyftlq

#26 aa320-344 kkpldgeyftlqirgrerfemfrel

#27 aa333-357 rgrerfemfrelnealelkdaqagk

#28 aa346-370 ealelkdaqagkepggsrahsshlk

#29 aa359-383 pggsrahsshlkskkgqstsrhkkl

#30 aa372-393 kkgqstsrhkklmfktegpdsd
