# Supplementary material for: Split T Cell Tolerance against a Self/Tumor Antigen: Spontaneous CD4+ but Not CD8+ T Cell Responses against p53 in Cancer Patients and Healthy Donors
Source: PLoS One. 2011 Aug 12;6(8):e23651. doi: 10.1371/journal.pone.0023651 (PMC3155555; doi:10.1371/journal.pone.0023651)
Supplement: Table S2 — Spontaneous immune responses against tumor-related antigens investigated in the New York Branch of Ludwig Institute for Cancer Research. (DOC) [file pone.0023651.s004.doc]

**Supplemental Table S2**

Spontaneous immune responses against tumor-related antigens investigated in the New York Branch of Ludwig Institute for Cancer Research.

Antigens Expression in Spontaneous immune response^a^

(references) adult normal tissues Antibody CD8^+^ T cells CD4^+^ T cells

P^b^ H^b^ SPP^c^ SNP+H^c^ SPP^c^ SNP+H^c^

NY-ESO-1 (1,2) Testis +++ - +++ - +++ -

p53 (this study) Ubiquitous +++ - - - +++ +++

NY-CO-58 (3) Testis, Others (low levels) -^d^ - NA - NA +++

MAGE-A3 (4,5) Testis ++ - -^e^ - +++ -

^a^ -:0-5%; +: 5-10%; ++: 10-30%; +++: >30%. NA: not available.

^b^ P: Cancer patients; H: Healthy individuals.

^c^ SPP: Seropositive cancer patients; SNP+H: Seronegative patients and healthy individuals.

^d^ NY-CO-58 was originally discovered by SEREX but has yet to be successfully confirmed by ELISA against recombinant protein.

^e^ CD8^+^ T cell responses were detected transiently in a few patients vaccinated with MAGE-A3 protein in AS02B.

**References**

(1) Jager, E., Y. Nagata, S. Gnjatic, H. Wada, E. Stockert, J. Karbach, P. R. Dunbar, S. Y. Lee, A. Jungbluth, D. Jager, M. Arand, G. Ritter, V. Cerundolo, B. Dupont, Y. T. Chen, L. J. Old, and A. Knuth. 2000. Monitoring CD8 T cell responses to NY-ESO-1: correlation of humoral and cellular immune responses. *Proc. Natl. Acad. Sci. USA* 97:4760-4765.

(2) Gnjatic, S., D. Atanackovic, E. Jager, M. Matsuo, A. Selvakumar, N. K. Altorki, R. G. Maki, B. Dupont, G. Ritter, Y. T. Chen, A. Knuth, and L. J. Old. 2003. Survey of naturally occurring CD4+ T cell responses against NY-ESO-1 in cancer patients: correlation with antibody responses. *Proc Natl Acad Sci U S A* 100:8862-8867.

(3) Gnjatic, S., Y. Cao, U. Reichelt, E. F. Yekebas, C. Nolker, A. H. Marx, A. Erbersdobler, H. Nishikawa, Y. Hildebrandt, K. Bartels, C. Horn, T. Stahl, I. Gout, V. Filonenko, K. L. Ling, V. Cerundolo, T. Luetkens, G. Ritter, K. Friedrichs, R. Leuwer, S. Hegewisch-Becker, J. R. Izbicki, C. Bokemeyer, L. J. Old, and D. Atanackovic. 2010. NY-CO-58/KIF2C is overexpressed in a variety of solid tumors and induces frequent T cell responses in patients with colorectal cancer. *Int J Cancer* 127:381-393.

(4) Tsuji, T., N. K. Altorki, G. Ritter, L. J. Old, and S. Gnjatic. 2009. Characterization of preexisting MAGE-A3-specific CD4+ T cells in cancer patients and healthy individuals and their activation by protein vaccination. *J Immunol* 183:4800-4808.

(5)Atanackovic, D., N. K. Altorki, Y. Cao, E. Ritter, C. A. Ferrara, G. Ritter, E. W. Hoffman, C. Bokemeyer, L. J. Old, and S. Gnjatic. 2008. Booster vaccination of cancer patients with MAGE-A3 protein reveals long-term immunological memory or tolerance depending on priming. *Proc Natl Acad Sci U S A* 105:1650-1655.
